# Patellar Tendon Shear Wave Velocity Is Higher and has Different Regional Patterns in Elite Competitive Alpine Skiers than in Healthy Controls

**DOI:** 10.3389/fbioe.2022.858610

**Published:** 2022-06-09

**Authors:** Tobias Götschi, Jonas Hanimann, Nicole Schulz, Simon Huser, Victoria Held, Walter O. Frey, Jess G. Snedeker, Jörg Spörri

**Affiliations:** ^1^ Department of Orthopedics, Balgrist University Hospital, University of Zurich, Zurich, Switzerland; ^2^ Institute for Biomechanics, ETH Zurich, Zurich, Switzerland; ^3^ Sports Medical Research Group, Department of Orthopedics, Balgrist University Hospital, University of Zurich, Zurich, Switzerland; ^4^ University Centre for Prevention and Sports Medicine, Balgrist University Hospital, University of Zurich, Zurich, Switzerland

**Keywords:** knee, tendinopathy, shear wave elastography, injury prevention, athletes, alpine ski racing

## Abstract

Competitive alpine skiers are exposed to enormous forces acting on their bodies–particularly on the knee joint and hence the patellar tendon - during both the off-season preparation and in-season competition phases. However, factors influencing patellar tendon adaptation and regional pattern differences between alpine skiers and healthy controls are not yet fully understood, but are essential for deriving effective screening approaches and preventative countermeasures. Thirty elite competitive alpine skiers, all members of the Swiss Alpine Ski Team, and 38 healthy age-matched controls were recruited. A set of two-dimensional shear wave elastography measurements of the PT was acquired and projected into three-dimensional space yielding a volumetric representation of the shear wave velocity profile of the patellar tendon. Multivariate linear models served to quantify differences between the two cohorts and effects of other confounding variables with respect to regional shear wave velocity. A significant (*p* < 0.001) intergroup difference was found between skiers (mean ± SD = 10.4 ± 1.32 m/s) and controls (mean ± SD = 8.9 ± 1.59 m/s). A significant sex difference was found within skiers (*p* = 0.024), but no such difference was found in the control group (*p* = 0.842). Regional SWV pattern alterations between skiers and controls were found for the distal region when compared to the mid-portion (*p* = 0.023). Competitive alpine skiers exhibit higher SWV in all PT regions than healthy controls, potentially caused by long-term adaptations to heavy tendon loading. The presence of sex-specific differences in PT SWV in skiers but not in controls indicates that sex effects have load-dependent dimensions. Alterations in regional SWV patterns between skiers and controls suggest that patellar tendon adaptation is region specific. In addition to the implementation of 3D SWE, deeper insights into long-term tendon adaptation and normative values for the purpose of preventative screening are provided.

## 1 Introduction

Tendons transmit the forces from muscles to the skeleton and are thus crucial for human locomotion. Similar to other connective tissues, tendons are able to respond to various intrinsic and extrinsic stimuli through morphological and material property adaptations. Among others, tendon loading, age (Carroll et al., 2008; Hsiao et al., 2015), sex (Magnusson et al., 2007), body mass index (BMI) (Gumina et al., 2014) and nutritional habits (Scott et al., 2015; Korntner et al., 2017) are factors influencing tendon properties.

Repetitive exertion of tensile stress on tendons can promote tendon remodeling and thickening (Bohm et al., 2015), involving both collagen synthesis and degradation (Magnusson et al., 2010). Additionally, upregulated enzymatic cross-linking increases the structural integrity of the tissue (Svensson et al., 2016; Passini et al., 2021). Repetitive excessive overloading and inadequate repair mechanisms can, however, induce cumulated tendon damage and trigger degenerative pathological processes (Cook and Purdam, 2009), including an increase in collagen type III fibers, fibrocartilaginous changes through the decomposition of glycosaminoglycans (GAGs), neovascularization and tenocyte rounding and proliferation (Maffulli et al., 2000; Fredberg and Stengaard-Pedersen, 2008; Attia et al., 2014; Snedeker and Foolen, 2017). Compared to collagen type I fibers, collagen type III fibers exhibit a reduced ability to form cross-links, thus decreasing fiber orientation and mechanical strength within the tendon (Maffulli et al., 2000). GAGs contain highly hydrophilic side chains and therefore increase the water content within the tendon. These degenerative adaptations deteriorate the mechanical properties of the pathologic tendons (Seo et al., 2015; Ooi et al., 2016; Finnamore et al., 2019) and favor traumatic tendon injuries (Cook and Purdam, 2009; Yasui et al., 2017). From a screening and injury prevention perspective, however, both physiological and pathological processes are crucial since tendon pathologies move along a partially reversible continuum (Cook and Purdam, 2009).

Physiological adaptations contribute to athletic performance (energy storage-releasing capacity of tendon) (Mcguigan et al., 2006; Van Hooren and Zolotarjova, 2017; Groeber et al., 2021), whereas pathological developments can jeopardize an athletic career (Kettunen et al., 2002; Lian et al., 2005; Zwerver et al., 2011). Transferring the forces exerted by M. quadriceps femoris from the lateral apex of the patella to the tibial tuberosity and, hence, being involved in many daily and sports-related activities, the patellar tendon (PT) bears high volumes of heavy loads. Substantial differences in morphological and mechanical PT properties between team sports athletes (i.e., Volleyball and basketball players) and the general population were reported (Zhang et al., 2015; Bayliss et al., 2016). In particular, high magnitude loading, such as exercising close to the maximal strength capacities, induced significant tendon adaptation (Malliaras et al., 2013; Bohm et al., 2015).

One cohort exposed to high PT loads during both the off-season preparation and in-season competition phases are competitive alpine skiers. During the preparation phase, plyometric and heavy weight exercises are frequently performed, both exerting large loads on the PT (Hydren et al., 2013). On-snow training and competition can require skiers to perform 33,000 turns per season, whereby the ground reaction force peaks at up to 4 times body weight (Supej and Holmberg, 2010; Gilgien et al., 2018). These forces act–due to the constantly flexed knee position–strongly on the knee extensor muscle-tendon unit and, thus, the PT (Alhammoud et al., 2020). Therefore, healthy competitive alpine skiers are considered a suitable cohort for the detection of physiological PT adaptations. However, it is not yet clear whether differences in tendon adaptation can be observed between competitive alpine skiers and healthy controls and to what extent other factors act on tendon properties within the two groups.

Ultrasound (US) shear wave elastography allows on-site assessment of tissue mechanical properties. A focused acoustic radiation impulse induces tissue displacement that propagates perpendicularly to the direction of the impulse and can be observed with high frame rate (3–18 kHz) brightness mode (B-mode) US and appropriate tracking algorithms (Hein and O’brien, 1993). The instantaneous group shear wave velocity (SWV) can be mapped on a regular grid superimposed onto the B-mode US image and is related to the tissues’ elastic properties (Nightingale, 2011). In detecting pathological and traumatic conditions of musculoskeletal structures, the quantitative assessment of elastic properties has proven valuable (Taljanovic et al., 2017). Particularly in depicting tendon damage and degeneration as well as predicting impeding structural failure, shear wave elastography (SWE) shows great potential (Aubry et al., 2015).

Tissue property assessments using US SWE are commonly performed in a two-dimensional (2D) manner. In particular, the investigation of larger structures is impeded by the limited field of view (FOV); therefore, the results are highly position dependent. In particular, tendon imaging requires accurate spatial referencing, as pathological characteristics in structural composition and architecture are typically spatially confined (Johnson et al., 1996; Khan et al., 1996; Pearson and Hussain, 2014). To overcome these limitations, we have previously implemented and validated an approach for three-dimensional US shear wave velocity mapping (Götschi et al., 2021). The technique allows sampling of larger structures primarily limited by the maximum measurement depth of the system and enables off-line analysis of distinct subregions (Götschi et al., 2021).

Based on this background, the current study was undertaken to 1) investigate differences in PT SWV in competitive alpine skiers compared to age-matched healthy control subjects, to 2) evaluate sex-specific differences in PT SWV, and to 3) describe potential differences in regional SWV patterns in both cohorts. Considering the literature and the high training volumes and loads in competitive alpine skiers, an increased SWV in the skier cohort compared to the control group was hypothesized. For the reasons stated above, it was also expected that the PT of male subjects would have a higher SWV than that of female subjects.

## Materials and Methods

### Participants and Study Design

Thirty-nine elite competitive alpine skiers (skier group), all members of the Swiss Alpine Ski Team, and 38 age-matched healthy controls were recruited for the conduction of this case–control study. In winter, i.e., during the competition period, the training of elite competitive alpine skiers typically includes 1 to 3 on-snow competitions and/or on-snow training sessions, as well as 7 to 14 off-snow training sessions per week (Gilgien et al., 2018). In summer, i.e., during the off-season training period, they train an average of 5–9 sessions on-snow and 6 to 18 sessions off-snow per week, resulting in a total of 14–21 h of training per week (Gilgien et al., 2018). During the first part of the off-season training period, in which also the present study was conducted, there is a particular focus on physical conditioning with 2–4 sessions of endurance training, 2–4 sessions of strength training, 1,2 sessions of explosive strength training and plyometrics, 2,3 sessions of agility and motor training, 3–5 sessions of stability and mobility training, and 1,2 sessions of cross training in other sports (Gilgien et al., 2018). The following inclusion criterion was applied: Physically active defined as more than one session of intense physical activity or more than 30 min of moderate physical activity per week. The number of participants recruited was limited by the number of active national competitive alpine skiers as part of the Swiss Alpine Ski Team. All skiers were monitored for health problems in a two-weekly interval with the Oslo Trauma Research Center (OSTRC) questionnaire for a minimum of 9 months prior to and up to the day of testing. Exclusion criteria were: 1) manifested signs of tendon disease or systemic pathology relevant to the purpose of the study; 2) acute PT tendon complaints; or 3) “severe” traumatic knee injuries leading to long-term training absences of more than 28 days (Fuller et al., 2006) within the 9 months preceding the US assessments. The medical records were verified and updated during an additional check-up by a medical doctor prior to SWV assessments. Nine subjects from the skier group were excluded, as they met exclusion criteria; 4 were excluded due to exclusion criteria 2, and 5 were excluded due to exclusion criteria 3. SWE assessments in the skier group were performed at the start of the off-season training period in May. Controls were particularly recruited to be healthy and not having experienced patellar tendon-related knee pain within the last 12 months. All subjects were informed of the aim of the study and provided written consent. The study was approved by the local ethics committee (KEK-ZH-NR: 2017-01395).

### Ultrasound Assessment

**FIGURE 1:**
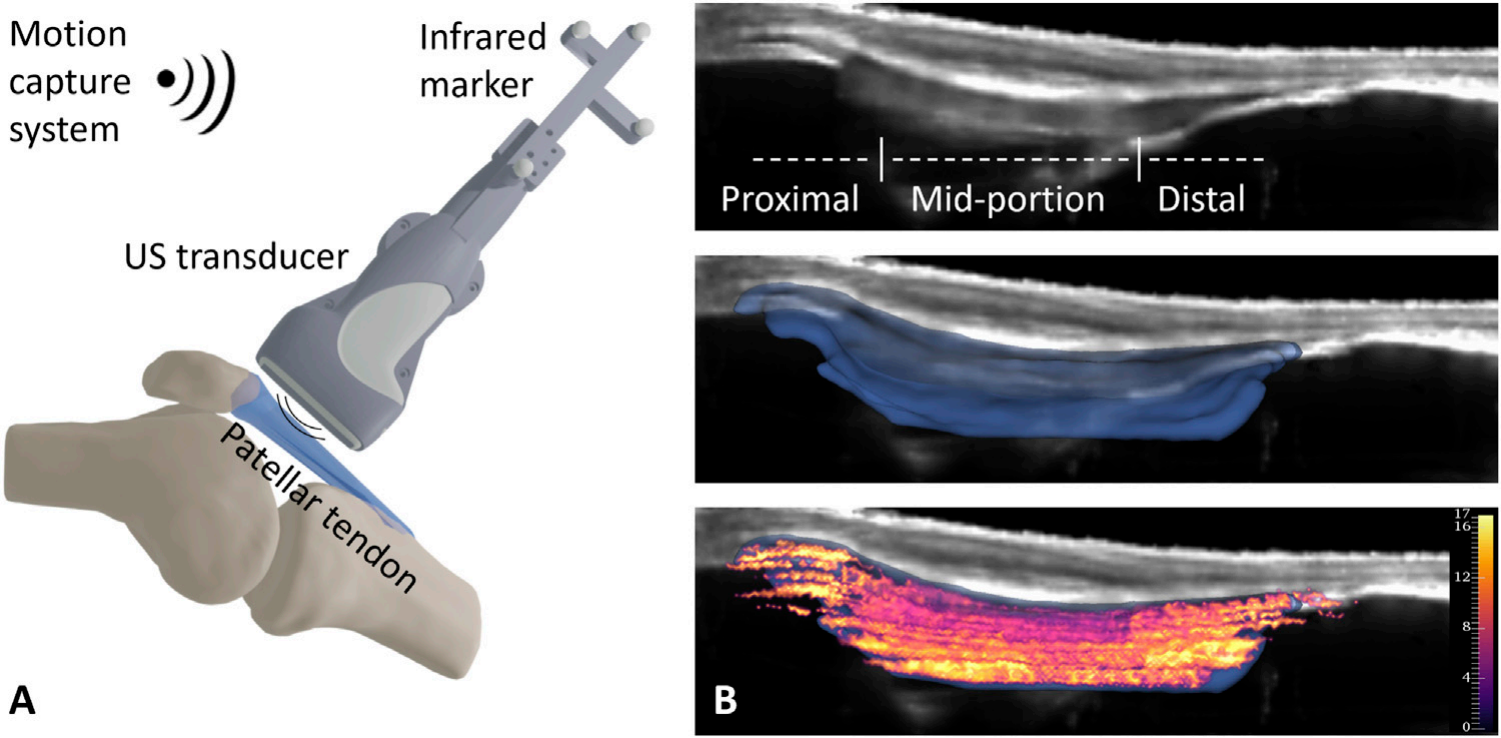
Schematic depiction of the ultrasound shear wave elastography analysis. **(A)** Ultrasound B-mode and shear wave elastography measurements were acquired with a linear ultrasound probe equipped with optical markers. **(B)** Two-dimensional measurements were projected into three-dimensional space to yield volumetric B-mode and shear wave elastography representations (top image). The patellar tendon was segmented (middle image), and the segmentation was applied to the shear wave elastography volume (bottom image).

US assessments and data processing were performed exactly as described in our validation study, which revealed *good* interoperator reliability (ICC of 0.736) and *very good* interday reliability (ICC of 0.904) (Götschi et al., 2021). One assessment was performed for each tendon by an experienced examiner; in total, 4 examiners were involved. The participant was in the supine position with adjustable support underneath the knee and a knee flexion angle of approximately 20°, and the right patellar tendon was examined (Martinoli, 2010). A US device (Aixplorer^®^ Ultimate, Supersonic Imagine, Aix-en-Provence, France) capable of high frame-rate US acquisition equipped with a linear transducer (SL18-5) was used. The US transducer was held perpendicular to the skin, and parallel alignment with the fiber axis was ensured by observing and maximizing the fibrillar appearance of the intratendinous structure. Position and orientation of the US transducer was tracked throughout the scanning procedure with optical markers attached to it and an optical tracking system (FusionTrack 500, Atracsys LLC) (Figure 1A). 2D SWE and US brightness-mode (B-mode) data were projected into 3D space and aggregated using a weighted averaging scheme to yield a 3D voxel representation of the measurement (MATLAB 2020a; The MathWorks, Inc., Natick, MA, United States ). The tendon was then segmented in the US B-mode volume, and the SWE volume was masked accordingly (3D slicer, version 4.10.2, (Kikinis et al., 2014). To allow analysis of distinct PT regions, the masked SWE reconstruction was divided into three regions with two cuts positioned at a distance of 10 mm from the proximal and distal insertion (Figure 1B), analogous to the approach described in our validation study (Götschi et al., 2021). Briefly, the area of interest was identified using landmarks placed during manual segmentation of the volume. Accordingly, the most distal point of attachment of the patella to the patellar tendon and the most proximal point of attachment of the tibia were marked for the proximal and distal sections of the PT. Further analysis was performed based on mean values for the respective regions.

### Statistical Analysis

To estimate the association of group membership while correcting for other potentially confounding variables with regional SWV, a multivariate approach is needed. Given the sample size available, including all factors of interest (including interaction effects) in one linear model may yield erroneous estimates due to overfitting. Hence, we opted for an alternative approach where we determined the linear model for inference by selecting the set of predictors with the best predictive performance in a leave-one-out validation scheme (maximum adjusted *R* ^2^). Consequently, for factors not included in the final models, no effect estimates are available. Univariate comparisons between groups were conducted with independent samples t tests. Statistical analysis was performed with R (Team, 2021), and statistical significance was set at *α* = 0.05.

## Results

### Baseline Characteristics

The baseline characteristics of both study groups are summarized in Table 1.

**TABLE 1:** Baseline characteristics.

*Parameter*	*Control Group (n = 38, female: 20)*	*Skier Group (n = 30, female: 14)*	*Overall (n = 68, female: 34)*	*p value*	
*Age [years]*	24.9 ± 2.50	23.6 ± 2.50	24.4 ± 2.57	0.982	ns
*Weight [kg]*	65.9 ± 10.3	73.7 ± 10.7	69.3 ± 11.1	0.002	**
*Height [cm]*	172 ± 8.71	173 ± 9.37	172 ± 8.95	0.324	ns
*BMI [kg/m* ^*2*^ *]*	22.2 ± 2.18	24.5 ± 1.81	23.2 ± 2.32	<0.001	**

Data are expressed as the mean ± SD (**): *p* < 0.01 (ns): *p* > 0.05.

### Overall Study Population

Linear regression modeling revealed group membership to be the only determining factor of SWV over the entire study population for all assessed tendon regions (overall, proximal, mid-portion, and distal), with skiers displaying higher SWV (Table 2).

**TABLE 2:** Inference models identified group membership as the only significant predictor for shear wave velocity of the entire study population in all assessed tendon regions.

*Tendon region*	*Predictor*	*Estimate [ms* ^*−1*^ *]*	*Std-Error [ms* ^*−1*^ *]*	*p value*	
*Overall*	Skier group	1.495	0.328	< 0.001	**
	Sex	0.263	0.325	0.422	ns
*Proximal*	Skier group	1.184	0.357	0.002	**
	Sex	0.116	0.366	0.753	ns
	Tendon length	0.054	0.028	0.061	ns
*Mid-portion*	Skier group	1.401	0.359	< 0.001	**
	Sex	0.256	0.365	0.486	ns
	Tendon length	0.045	0.028	0.117	ns
*Distal*	Skier group	2.202	0.447	< 0.001	**
	Sex	0.023	0.443	0.959	ns

(**): *p* < 0.01, (ns): *p* > 0.05.

### Predictive Factors of Shear Wave Velocity in Each Study Group

When assessing each study group separately, in the skier group, both subject age and sex were retained in the final regression model for the midportion region of the PT (Table 3). In the control group, no predictors for mid-portion SWV were identified.

**TABLE 3:** Predictive factors of shear wave velocity in the skier group. In the control group, no predictive factors were identified.

Target	Predictor	Estimate	Std- error	*p* value		
Skier Group						
Proximal	Male sex	0.927 ms-1	0.524 ms-1	0.088		ns	
	Tendon length	0.058	0.034	0.102		ns	
	BMI	−0.212	0.141	0.144		ns	
Mid-portion	Age	−0.172 ms-1year-1	0.087 ms-1year-1	0.057		ns	
	Male sex	1.080 ms-1	0.428 ms-1	0.018		*	
Distal	Male sex	0.035 ms-1	0.678 ms-1	0.960		ns	

(*): *p* < 0.05, (ns): *p* > 0.05.

**FIGURE 2:**
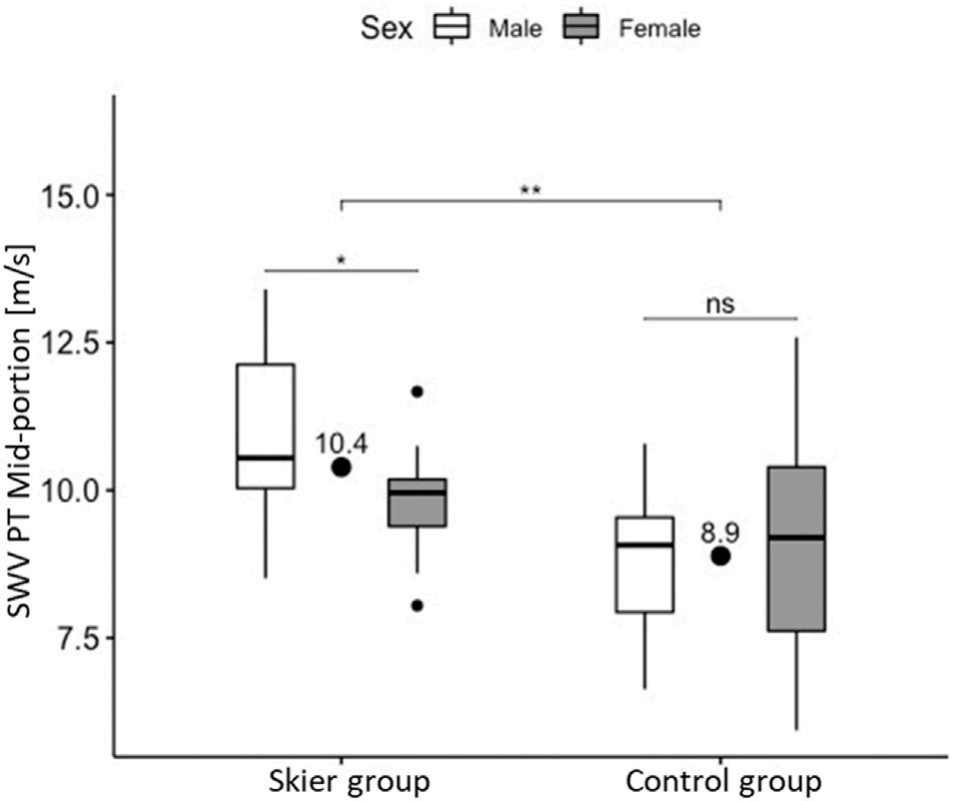
Shear wave velocity of the midportion of the patellar tendon as a function of group membership and sex. (•): Group averages. Box: Median, interquartile range, range and outliers (*): *p* < 0.05 (**): *p* < 0.01 (ns): *p* > 0.05.

Figure 2 visualizes shear wave velocity differences between groups as well as the significant sex effect in the skier group and the absence thereof in the control group.

### Regional Shear Wave Velocity Patterns in Patellar Tendons

**FIGURE 3:**
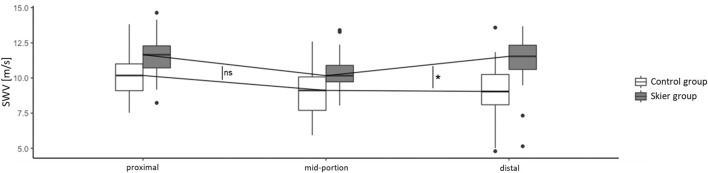
Regional shear wave velocity in both study groups. Box: Median, interquartile range, range and outliers.

We found that the regional difference in SWV between the distal and mid-portion PTs differed between the skier and control groups (*p* = 0.023). The two groups, however, did not differ with regard to the regional delta between the proximal and PT mid-portions (*p* = 0.599, Figure 3).

## Discussion

This study found higher PT SWV in competitive alpine skiers than in healthy controls. No demographic factors were predictive of SWV in the entire study cohort. Male skiers had a higher SWV than female skiers, whereas no such difference was found in the control group. Furthermore, skiers displayed a larger SWV decrease from the distal tendon to its mid-portion.

### Higher Shear Wave Velocity in Competitive Alpine Skiers Than Healthy Controls

Physiological tendon hypertrophy and remodeling in response to altered loading regimes allows the tendon to cope with increased training volume and intensity. Moreover, optimization of muscle tendon unit performance requires both muscle and tendon to adapt, whereas the tendon is responsible for effective force transmission. In this context, lower tendon hysteresis has been reported in ski jumpers and runners compared to control subjects (Wiesinger et al., 2017). Pathological adaptations, on the other hand, lead to deterioration of the tendon structure, limiting the capacity for physical performance and increasing the risk of tendon rupture (Cook and Purdam, 2009; Ooi et al., 2016; Yasui et al., 2017). Competitive alpine skiers are exposed to tremendous loads both during off-season training and in the competition season (Supej and Holmberg, 2010; Hydren et al., 2013; Gilgien et al., 2018). High accelerations combined with forward pushed, highly flexed knees induce large forces acting on the knee joint and, in particular, the PT (Alhammoud et al., 2020). Moreover, compared to other sports, particularly in competition situations the demands on the PT in alpine skiers are relatively high (90–120 s of high-intensity loading of the quadriceps muscles with peak ground reaction forces of up to four times body weight) (Supej and Holmberg, 2010). Therefore, off-snow training modalities are also designed to meet these unique demands, making extensive adaptations of the PT in skiers theoretically sound. Finally, such extraordinary demands can overwhelm the system’s capacity for adaptation, which may manifest in a high prevalence of overuse complaints in this cohort (Fröhlich et al., 2020; Fröhlich et al., 2021). A closer understanding of the adaptive processes in response to long-term repetitive loading with regard to PT material properties is crucial in prevention and early detection strategies.

### Influence of Demographic Factors

When assessing intercohort differences, adjustment of cofounding variables such as demographic factors is necessary. To do so, the selection of linear models with the best predictive performance is a suitable method (Stone, 1974). In our dataset, the effect of the tendon loading regime (i.e., group membership) outweighed biological factors such as weight, height, sex, age, tendon CSA, and tendon length. Zhang et al. reported lower stiffness in the PT of athletes; however, the inclusion criteria for the athletic cohort was a minimum of 4 h of exercise per week; hence, the inclusion criteria for the control and athlete groups are not comparable with our study (Zhang et al., 2015). Other studies agree with our finding: Increased PT SWV in athletes has been reported when compared to nonathletic cohorts (Zhang et al., 2015; Bayliss et al., 2016; Mersmann et al., 2017; Cristi-Sanchez et al., 2019; Dirrichs et al., 2019; Selcuk Can et al., 2021). A quantitative comparison of relative magnitude from our study shows a 1.17 times higher SWV in PT in skiers compared to the control group, whereas it is 1.47 when calculated with the results from Selcuk Can et al. (Selcuk Can et al., 2021). The difference may be explained by the different activity levels of the control and athlete groups in the two studies, but nevertheless, the two factors are in a similar range.

### Potential Influence of Different Loading Regimes

Furthermore, increased tendon SWV values have been reported to result from altered loading regimes (Malliaras et al., 2013; Bohm et al., 2015), confirming the tendon’s ability to adapt to load and supporting our finding that the loading regime is the main factor inducing physiological tendon adaptation. Interestingly, a sex-specific difference in SWV was only found in the skier group, which may indicate that high loads must be present to elicit sex-specific adaptation mechanisms. Some studies suggest that there is a sex-specific difference in mechanical tendon properties (Kubo et al., 2003; Onambele et al., 2007; Tas et al., 2017), while others do not (Burgess et al., 2009; O’brien et al., 2010; Morrison et al., 2015); hence, in the literature, there is no consensus in this regard. On the one hand, hormone-based effects on tendon adaptation have been described: Acute exercise-induced increase of tendon collagen synthesis is inhibited by estrogen (Magnusson et al., 2007) and is therefore diminished in magnitude and time, compared with men (Miller et al., 2007). On the other hand, male subjects are both heavier and suspected to be able to generate higher knee extension moments than female subjects, leading to increased PT forces and subsequently inducing PT adaptation. In agreement with this hypothesis, Arampatzis et al. found a positive correlation between maximal knee extension moment and PT stiffness (Arampatzis et al., 2007). Another study found increased PT stiffness in heavy loaded legs, whereas no effect was found in light loaded legs (Kongsgaard et al., 2007). Furthermore, no significant correlation between knee extension torque and PT stiffness has been found in subjects with no specific training engagement (Mannarino et al., 2018), thus supporting our hypothesis. The literature states that female sex is a protective factor regarding patellar tendinopathies, referring to team sport athletes (Morton et al., 2017). Since the difference in PT SWV was observed in competitive alpine skiers but not in the control group, it may be assumed that lower forces applied in female recreational athletes and, therefore, overuse complaints at the PT occur less frequently.

### Differences in Regional Patellar Tendon Shear Wave Velocity

In the current study, different regional patterns in the PT SWV were found between skiers and controls, with significant differences between the delta from the mid-portion to the distal region. Functionally and clinically, proximal regions, mid-portions, and distal regions differ, and thus, it is expected that SWV as a quantification for tendon adaptation (i.e., collagen cross-linking) reflects the heterogeneous regional characteristics. Interestingly, Kongsgaard and others found an increase in proximal PT CSA after light and heavy training in healthy untrained men but a distal increase in CSA only after heavy training, with the training consisting of knee extension exercise (Kongsgaard et al., 2007). Since our study included only physically active subjects and yet the athletic cohort performs heavier physical activity, our finding is reflected by the results from Kongsgaard et al. (Kongsgaard et al., 2007). The precise structural adaptations that underlie elevated tendon SWV are currently unclear; nevertheless, the nonuniform adaptation behavior along the PT in response to the loading regime cannot be neglected. Thus, further research should implement a more sophisticated view regarding loading magnitude with regard to regional adaptations.

### Study Limitations

This study has limitations to be considered. Physical activity levels of control subjects were not quantified on a continuous scale, and thus, there are potentially highly active subjects included in the control group. Since high-level athletes usually have more muscle mass than recreational athletes, BMI between these groups significantly differs, hence representing a cofounding factor. Furthermore, a challenge in assessing tendon SWV is the alignment between the transducer and tendon fiber orientation. Despite greatest caution in aligning the transducer to the fiber orientation, situations where the fibers are not colinear over the entire measurement frame cannot be compensated for. Finally, tendon loading in the hours prior to or during the measurement was not controlled for; therefore, we asked the subjects to remain relaxed in the measurement position for 5 minutes and refrain from strenuous physical activity 24 h prior to the assessment. An interesting aspect that cannot be concluded from the present study and requires further investigation is whether there are differences in the SWV profiles of skiers in the different periods of a season.

## Conclusion

Competitive alpine skiers showed higher SWV values in PT than physically active control subjects. The tendon loading regime is likely a main cause for the observed adaptations. Within skiers, males showed higher SWV values than females in the control group; however, no sex-specific difference was found. Hence, the intracohort analysis indicates that vast loading magnitudes and volumes are required to elicit such effects. Differences in the regional SWV pattern in the two cohorts indicate PT tissue adaptations to be dependent on the anatomical location. Translating our findings toward clinical applications and prevention strategies, screening and appropriate strength exercise might represent a fundamental component; however, normative data for different cohorts are fundamental in turns of a meaningful screening program.

## Data Availability

The datasets presented in this article are not readily available because their access is restricted to protect the interests of the project partner Swiss-Ski and their athletes. Requests to access the datasets should be directed to joerg.spoerri@balgrist.ch.

## References

[B1] Alhammoud M. Hansen C. Meyer F. Hautier C. Morel B. (2020). On-Field Ski Kinematic According to Leg and Discipline in Elite Alpine Skiers. Front. Sports Act. Living 2, 56. 10.3389/fspor.2020.00056 33345047 PMC7739787

[B2] Arampatzis A. Karamanidis K. Morey-Klapsing G. De Monte G. Stafilidis S. (2007). Mechanical Properties of the Triceps Surae Tendon and Aponeurosis in Relation to Intensity of Sport Activity. J. Biomechanics 40, 1946–1952. 10.1016/j.jbiomech.2006.09.005 17101142

[B3] Attia M. Scott A. Carpentier G. Lian Ø. Van Kuppevelt T. Gossard C. (2014). Greater Glycosaminoglycan Content in Human Patellar Tendon Biopsies Is Associated with More Pain and a Lower VISA Score. Br. J. Sports Med. 48, 469–475. 10.1136/bjsports-2013-092633 24100290

[B4] Aubry S. Nueffer J.-P. Tanter M. Becce F. Vidal C. Michel F. (2015). Viscoelasticity in Achilles Tendonopathy: Quantitative Assessment by Using Real-Time Shear-Wave Elastography. Radiology 274, 821–829. 10.1148/radiol.14140434 25329764

[B5] Bayliss A. J. Weatherholt A. M. Crandall T. T. Farmer D. L. Mcconnell J. C. Crossley K. M. (2016). Achilles Tendon Material Properties Are Greater in the Jump Leg of Jumping Athletes. J. Musculoskelet. Neuronal Interact. 16, 105–112. 27282454 PMC5114353

[B6] Bohm S. Mersmann F. Arampatzis A. (2015). Human Tendon Adaptation in Response to Mechanical Loading: a Systematic Review and Meta-Analysis of Exercise Intervention Studies on Healthy Adults. Sports Med. - Open 1, 7. 10.1186/s40798-015-0009-9 27747846 PMC4532714

[B7] Burgess K. E. Pearson S. J. Breen L. Onambélé G. N. L. (2009). Tendon Structural and Mechanical Properties Do Not Differ between Genders in a Healthy Community-Dwelling Elderly Population. J. Orthop. Res. 27, 820–825. 10.1002/jor.20811 19058184

[B8] Carroll C. C. Dickinson J. M. Haus J. M. Lee G. A. Hollon C. J. Aagaard P. (20081985). Influence of Aging on the *In Vivo* Properties of Human Patellar Tendon. J. Appl. Physiology 105, 1907–1915. 10.1152/japplphysiol.00059.2008 PMC2612460 18927271

[B9] Cook J. L. Purdam C. R. (2009). Is Tendon Pathology a Continuum? A Pathology Model to Explain the Clinical Presentation of Load-Induced Tendinopathy. Br. J. Sports Med. 43, 409–416. 10.1136/bjsm.2008.051193 18812414

[B10] Cristi-Sánchez I. Danes-Daetz C. Neira A. Ferrada W. Yáñez Díaz R. Silvestre Aguirre R. (2019). Patellar and Achilles Tendon Stiffness in Elite Soccer Players Assessed Using Myotonometric Measurements. Sports Health 11, 157–162. 10.1177/1941738118820517 30601077 PMC6391548

[B11] Dirrichs T. Schrading S. Gatz M. Tingart M. Kuhl C. K. Quack V. (2019). Shear Wave Elastography (SWE) of Asymptomatic Achilles Tendons: A Comparison between Semiprofessional Athletes and the Nonathletic General Population. Acad. Radiol. 26, 1345–1351. 10.1016/j.acra.2018.12.014 30655054

[B12] Finnamore E. Waugh C. Solomons L. Ryan M. West C. Scott A. (2019). Transverse Tendon Stiffness Is Reduced in People with Achilles Tendinopathy: A Cross-Sectional Study. PLoS One 14, e0211863. 10.1371/journal.pone.0211863 30785895 PMC6382130

[B13] Fredberg U. Stengaard-Pedersen K. (2008). Chronic Tendinopathy Tissue Pathology, Pain Mechanisms, and Etiology with a Special Focus on Inflammation. Scand. J. Med. Sci. Sports 18, 3–15. 10.1111/j.1600-0838.2007.00746.x 18294189

[B14] Fröhlich S. Helbling M. Fucentese S. F. Karlen W. Frey W. O. Spörri J. (2021). Injury Risks Among Elite Competitive Alpine Skiers Are Underestimated if Not Registered Prospectively, over the Entire Season and Regardless of whether Requiring Medical Attention. Knee Surg. Sports Traumatol. Arthrosc. 29, 1635–1643. 10.1007/s00167-020-06110-5 32556431

[B15] Fröhlich S. Peterhans L. Stern C. Frey W. O. Sutter R. Spörri J. (2020). Remarkably High Prevalence of Overuse-Related Knee Complaints and MRI Abnormalities in Youth Competitive Alpine Skiers: a Descriptive Investigation in 108 Athletes Aged 13-15 Years. BMJ Open Sport Exerc Med. 6, e000738. 10.1136/bmjsem-2020-000738 PMC7264838 32537242

[B16] Fuller C. W. Ekstrand J. Junge A. Andersen T. E. Bahr R. Dvorak J. (2006). Consensus Statement on Injury Definitions and Data Collection Procedures in Studies of Football (Soccer) Injuries. Br. J. Sports Med. 40, 193–201. 10.1136/bjsm.2005.025270 16505073 PMC2491990

[B17] Gilgien M. Reid R. Raschner C. Supej M. Holmberg H.-C. (2018). The Training of Olympic Alpine Ski Racers. Front. Physiol. 9, 1772. 10.3389/fphys.2018.01772 30622477 PMC6308179

[B18] Götschi T. Schulz N. Snedeker J. G. Hanimann J. Franchi M. V. Spörri J. (2021). Three-Dimensional Mapping of Shear Wave Velocity in Human Tendon: A Proof of Concept Study. Sensors (Basel) 21, 1655. 10.3390/s21051655 33673664 PMC7957754

[B19] Groeber M. Stafilidis S. Baca A. (2021). The Effect of Stretch-Shortening Magnitude and Muscle-Tendon Unit Length on Performance Enhancement in a Stretch-Shortening Cycle. Sci. Rep. 11, 14605. 10.1038/s41598-021-94046-2 34272461 PMC8285374

[B20] Gumina S. Candela V. Passaretti D. Latino G. Venditto T. Mariani L. (2014). The Association between Body Fat and Rotator Cuff Tear: the Influence on Rotator Cuff Tear Sizes. J. Shoulder Elb. Surg. 23, 1669–1674. 10.1016/j.jse.2014.03.016 24906904

[B21] Hein I. A. O'brien W. D. (1993). Current Time-Domain Methods for Assessing Tissue Motion by Analysis from Reflected Ultrasound Echoes-A Review. IEEE Trans. Ultrason. Ferroelect., Freq. Contr. 40, 84–102. 10.1109/58.212556 18263161

[B22] Hsiao M.-Y. Chen Y.-C. Lin C.-Y. Chen W.-S. Wang T.-G. (2015). Reduced Patellar Tendon Elasticity with Aging: *In Vivo* Assessment by Shear Wave Elastography. Ultrasound Med. Biol. 41, 2899–2905. 10.1016/j.ultrasmedbio.2015.07.008 26304500

[B23] Hydren J. R. Volek J. S. Maresh C. M. Comstock B. A. Kraemer W. J. (2013). Review of Strength and Conditioning for Alpine Ski Racing. Strength & Cond. J. 35, 10–28. 10.1519/ssc.0b013e31828238be 23207888

[B24] Johnson D. P. Wakeley C. J. Watt I. (1996). Magnetic Resonance Imaging of Patellar Tendonitis. J. Bone Jt. Surg. Br. 78-B, 452–457. 10.1302/0301-620x.78b3.0780452 8636185

[B25] Kettunen J. A. Kvist M. Alanen E. Kujala U. M. (2002). Long-Term Prognosis for Jumper's Knee in Male Athletes: Prospective Follow-Up Study. Am. J. Sports Med. 30, 689–692. 10.1177/03635465020300051001 12239003

[B26] Khan K. M. Bonar F. Desmond P. M. Cook J. L. Young D. A. Visentini P. J. (1996). Patellar Tendinosis (Jumper's Knee): Findings at Histopathologic Examination, US, and MR Imaging. Victorian Institute of Sport Tendon Study Group. Radiology 200, 821–827. 10.1148/radiology.200.3.8756939 8756939

[B27] Kikinis R. Pieper S. D. Vosburgh K. G. (2014). “3D Slicer: A Platform for Subject-specific Image Analysis, Visualization, and Clinical Support,” in Intraoperative Imaging and Image-Guided Therapy. Editor Jolesz F. A. (New York, NY: Springer New York), 277–289. 10.1007/978-1-4614-7657-3_19

[B28] Kongsgaard M. Reitelseder S. Pedersen T. G. Holm L. Aagaard P. Kjaer M. (2007). Region Specific Patellar Tendon Hypertrophy in Humans Following Resistance Training. Acta Physiol. 191, 111–121. 10.1111/j.1748-1716.2007.01714.x 17524067

[B29] Korntner S. Kunkel N. Lehner C. Gehwolf R. Wagner A. Augat P. (2017). A High-Glucose Diet Affects Achilles Tendon Healing in Rats. Sci. Rep. 7, 780. 10.1038/s41598-017-00700-z 28396584 PMC5429625

[B30] Kubo K. Kanehisa H. Fukunaga T. (2003). Gender Differences in the Viscoelastic Properties of Tendon Structures. Eur. J. Appl. Physiol. 88, 520–526. 10.1007/s00421-002-0744-8 12560950

[B31] Lian Ø. B. Engebretsen L. Bahr R. (2005). Prevalence of Jumper's Knee Among Elite Athletes from Different Sports: a Cross-Sectional Study. Am. J. Sports Med. 33, 561–567. 10.1177/0363546504270454 15722279

[B32] Maffulli N. Ewen S. W. B. Waterston S. W. Reaper J. Barrass V. (2000). Tenocytes from Ruptured and Tendinopathic Achilles Tendons Produce Greater Quantities of Type III Collagen Than Tenocytes from Normal Achilles Tendons: An *In Vitro* Model of Human Tendon Healing. Am. J. Sports Med. 28, 499–505. 10.1177/03635465000280040901 10921640

[B33] Magnusson S. P. Hansen M. Langberg H. Miller B. Haraldsson B. Kjoeller Westh E. (2007). The Adaptability of Tendon to Loading Differs in Men and Women. Int. J. Exp. Pathol. 88, 237–240. 10.1111/j.1365-2613.2007.00551.x 17696904 PMC2517312

[B34] Magnusson S. P. Langberg H. Kjaer M. (2010). The Pathogenesis of Tendinopathy: Balancing the Response to Loading. Nat. Rev. Rheumatol. 6, 262–268. 10.1038/nrrheum.2010.43 20308995

[B35] Malliaras P. Kamal B. Nowell A. Farley T. Dhamu H. Simpson V. (2013). Patellar Tendon Adaptation in Relation to Load-Intensity and Contraction Type. J. Biomechanics 46, 1893–1899. 10.1016/j.jbiomech.2013.04.022 23773532

[B36] Mannarino P. Lima K. M. M. Fontenelle C. R. C. Matta T. T. De Salles B. F. Simão R. (2018). Analysis of the Correlation between Knee Extension Torque and Patellar Tendon Elastic Property. Clin. Physiol. Funct. Imaging 38, 378–383. 10.1111/cpf.12424 28707752

[B37] Martinoli C. (2010). Musculoskeletal Ultrasound: Technical Guidelines. Insights Imaging 1, 99–141. 10.1007/s13244-010-0032-9 23100193 PMC3481034

[B38] Mcguigan M. R. Doyle T. L. A. Newton M. Edwards D. J. Nimphius S. Newton R. U. (2006). Eccentric Utilization Ratio: Effect of Sport and Phase of Training. J. Strength Cond. Res. 20, 992–995. 10.1519/r-19165.1 17194252

[B39] Mersmann F. Charcharis G. Bohm S. Arampatzis A. (2017). Muscle and Tendon Adaptation in Adolescence: Elite Volleyball Athletes Compared to Untrained Boys and Girls. Front. Physiol. 8, 417. 10.3389/fphys.2017.00417 28670285 PMC5472702

[B40] Miller B. F. Hansen M. Olesen J. L. Schwarz P. Babraj J. A. Smith K. (2007). Tendon Collagen Synthesis at Rest and after Exercise in Women. J. Appl. Physiology 102, 541–546. 10.1152/japplphysiol.00797.2006 16990502

[B41] Morrison S. M. Dick T. J. M. Wakeling J. M. (2015). Structural and Mechanical Properties of the Human Achilles Tendon: Sex and Strength Effects. J. Biomechanics 48, 3530–3533. 10.1016/j.jbiomech.2015.06.009 PMC4592795 26159060

[B42] Morton S. Williams S. Valle X. Diaz-Cueli D. Malliaras P. Morrissey D. (2017). Patellar Tendinopathy and Potential Risk Factors. Clin. J. Sport Med. 27, 468–474. 10.1097/jsm.0000000000000397 28151759

[B43] Nightingale K. (2011). Acoustic Radiation Force Impulse (ARFI) Imaging: a Review. Cmir 7, 328–339. 10.2174/157340511798038657 PMC3337770 22545033

[B44] O'brien T. D. Reeves N. D. Baltzopoulos V. Jones D. A. Maganaris C. N. (2010). Mechanical Properties of the Patellar Tendon in Adults and Children. J. Biomech. 43, 1190–1195. 10.1016/j.jbiomech.2009.11.028 20045111

[B45] Onambélé G. N. L. Burgess K. Pearson S. J. (2007). Gender-specific *In Vivo* Measurement of the Structural and Mechanical Properties of the Human Patellar Tendon. J. Orthop. Res. 25, 1635–1642. 10.1002/jor.20404 17568426

[B46] Ooi C. C. Richards P. J. Maffulli N. Ede D. Schneider M. E. Connell D. (2016). A Soft Patellar Tendon on Ultrasound Elastography Is Associated with Pain and Functional Deficit in Volleyball Players. J. Sci. Med. Sport 19, 373–378. 10.1016/j.jsams.2015.06.003 26095373

[B47] Passini F. S. Jaeger P. K. Saab A. S. Hanlon S. Chittim N. A. Arlt M. J. (2021). Shear-stress Sensing by PIEZO1 Regulates Tendon Stiffness in Rodents and Influences Jumping Performance in Humans. Nat. Biomed. Eng. 5, 1457–1471. 10.1038/s41551-021-00716-x 34031557 PMC7612848

[B48] Pearson S. J. Hussain S. R. (2014). Region-specific Tendon Properties and Patellar Tendinopathy: a Wider Understanding. Sports Med. 44, 1101–1112. 10.1007/s40279-014-0201-y 24838651

[B49] Scott A. Zwerver J. Grewal N. De Sa A. Alktebi T. Granville D. J. (2015). Lipids, Adiposity and Tendinopathy: Is There a Mechanistic Link? Critical Review. Br. J. Sports Med. 49, 984–988. 10.1136/bjsports-2014-093989 25488953 PMC4518755

[B50] Selcuk Can T. Ozdemir S. Yilmaz B. K. (2021). Shear-Wave Elastography of Patellar Ligament and Achilles Tendon in Semiprofessional Athletes: Comparing with the Nonexercising Individuals. J. Ultrasound Med. (Online ahead of print). 10.1002/jum.15908 34862639

[B51] Seo J.-B. Yoo J.-S. Ryu J.-W. (2015). Sonoelastography Findings of Supraspinatus Tendon in Rotator Cuff Tendinopathy without Tear: Comparison with Magnetic Resonance Images and Conventional Ultrasonography. J. Ultrasound 18, 143–149. 10.1007/s40477-014-0148-8 26191102 PMC4504856

[B52] Snedeker J. G. Foolen J. (2017). Tendon Injury and Repair - A Perspective on the Basic Mechanisms of Tendon Disease and Future Clinical Therapy. Acta Biomater. 63, 18–36. 10.1016/j.actbio.2017.08.032 28867648

[B53] Stone M. (1974). Cross-Validatory Choice and Assessment of Statistical Predictions. J. R. Stat. Soc. Ser. B Methodol. 36, 111–133. 10.1111/j.2517-6161.1974.tb00994.x

[B54] Supej M. Holmberg H.-C. (2010). How Gate Setup and Turn Radii Influence Energy Dissipation in Slalom Ski Racing. J. Appl. Biomech. 26, 454–464. 10.1123/jab.26.4.454 21245505

[B55] Svensson R. B. Heinemeier K. M. Couppé C. Kjaer M. Magnusson S. P. (2016). Effect of Aging and Exercise on the Tendon. J. Appl. Physiol. (1985) 121, 1237–1246. 10.1152/japplphysiol.00328.2016 27150831

[B56] Taljanovic M. S. Gimber L. H. Becker G. W. Latt L. D. Klauser A. S. Melville D. M. (2017). Shear-Wave Elastography: Basic Physics and Musculoskeletal Applications. Radiographics 37, 855–870. 10.1148/rg.2017160116 28493799 PMC5452887

[B57] Tas S. Yilmaz S. Onur M. R. Soylu A. R. Altuntas O. Korkusuz F. (2017). Patellar Tendon Mechanical Properties Change with Gender, Body Mass Index and Quadriceps Femoris Muscle Strength. Acta Orthop. Traumatol. Turc 51, 54–59. 28010997 10.1016/j.aott.2016.12.003 PMC6197583

[B58] Team R. C. (2021). R: A Language and Environment for Statistical Computing. Vienna, Austria: R Foundation for Statistical Computing. Available at: https://www.R-project.org/.

[B59] Van Hooren B. Zolotarjova J. (2017). The Difference between Countermovement and Squat Jump Performances: A Review of Underlying Mechanisms with Practical Applications. J. Strength & Cond. Res. 31, 2011–2020. 10.1519/jsc.0000000000001913 28640774

[B60] Wiesinger H.-P. Rieder F. Kösters A. Müller E. Seynnes O. R. (2017). Sport-Specific Capacity to Use Elastic Energy in the Patellar and Achilles Tendons of Elite Athletes. Front. Physiol. 8, 132. 10.3389/fphys.2017.00132 28348529 PMC5346584

[B61] Yasui Y. Tonogai I. Rosenbaum A. J. Shimozono Y. Kawano H. Kennedy J. G. (2017). The Risk of Achilles Tendon Rupture in the Patients with Achilles Tendinopathy: Healthcare Database Analysis in the United States. Biomed. Res. Int. 2017, 7021862. 10.1155/2017/7021862 28540301 PMC5429922

[B62] Zhang Z. J. Ng G. Y. F. Fu S. N. (2015). Effects of Habitual Loading on Patellar Tendon Mechanical and Morphological Properties in Basketball and Volleyball Players. Eur. J. Appl. Physiol. 115, 2263–2269. 10.1007/s00421-015-3209-6 26126839

[B63] Zwerver J. Bredeweg S. W. Van Den Akker-Scheek I. (2011). Prevalence of Jumper's Knee Among Nonelite Athletes from Different Sports. Am. J. Sports Med. 39, 1984–1988. 10.1177/0363546511413370 21737835

